# Chemometric Methods for Simultaneous Determination of Candesartan Cilexetil and Hydrochlorothiazide in Binary Combinations

**DOI:** 10.1155/2023/5107317

**Published:** 2023-01-17

**Authors:** Diyar Salahuddin Ali

**Affiliations:** ^1^Department of Chemistry, College of Science, Salahaddin University, Kurdistan Region, Erbil, Iraq; ^2^Department of Medical Laboratory Science, College of Health Sciences, Lebanese French University, Kurdistan Region, Erbil, Iraq

## Abstract

Simple, accurate, precise, and cost-effective chemometric techniques for the measurement of candesartan cilexetil and hydrochlorothiazide in synthetic mixtures were improved and validated. *H*-point standard addition, *Q*-absorption ratio, and correction absorbance spectrophotometric techniques were utilized for the simultaneous determination of both medicines in real pharmaceutical formulations. A new calibration approach was implemented based on chemical *H*-point standards. This approach was developed to resolve significantly overlapping spectra of two analytes and provide direct correction of both proportional and constant errors caused by the matrix of the sample. The first method of simultaneous determination of candesartan cilexetil and hydrochlorothiazide was carried out using the *H*-point standard addition method at wavelengths 239 and 283. For the ratio of the absorption at two selected wavelengths, one of which is the isoabsorptive point and the other being the maximum of one of the two components, the second method absorption ratio method was utilized. In distilled water, the isoabsorptive point of candesartan cilexetil and hydrochlorothiazide occurs at 258 nm. *λ*_max_ of hydrochlorothiazide is 273 nm, which is the second wavelength used. Lastly, the absorbance correction method was implemented. This approach is based on absorbance correction equations and uses distilled water as the solvent for the examination of both medicines. In NaOH/EtOH solvent, the absorbance maxima of candesartan cilexetil and hydrochlorothiazide are 250 nm and 340 nm, respectively. For both wavelengths, candesartan cilexetil and hydrochlorothiazide exhibited linearity over a concentration range of 1–46 *μ*g/ml and 1–44 *μ*g/ml, respectively, for *H*-point standard addition. The *Q*-absorption ratio approach provides linearity over the concentration ranges of 1–46 *μ*g/ml at 273 nm for candesartan cilexetil and 1–29 *μ*g/ml for hydrochlorothiazide, 1–46 *μ*g/ml at 258 nm for candesartan cilexetil, and 1–44 *μ*g/ml for hydrochlorothiazide. For hydrochlorothiazide, the linearity for the correction absorbance method was obtained throughout a concentration range of 1–46 *μ*g/ml at wavelengths 250 and 340 nm and 1–44 *μ*g/ml at wavelength 250 nm. The results of the analysis have been statistically and empirically supported by recovery studies. All methods yielded recoveries in the range of 96 –102% for both medications. The LOD ranged from 0.46 –0.94 *μ*g/mL for hydrochlorothiazide and from 1.26 –2.40 *μ*g/mL for candesartan cilexetil. The approaches were then used to quantify candesartan cilexetil and hydrochlorothiazide in pharmaceutical tablets.

## 1. Introduction

Candesartan cilexetil (CAN) 2-ethoxy-1-((4-[2-(2H-1,2,3,4-tetrazol-5-yl) phenyl] phenyl, methyl))-1H-1, 3-benzodiazole-7-carboxylic acid is an angiotensin receptor blocker used mainly for the treatment of high blood pressure and congestive heart failure. Candesartan has a very low maintenance dose. Hydrochlorothiazide (HCT), 6-chloro-3,4-dihydro-2H-1,2,4-benzothiadiazine-7-sulfonamide-1,1-dioxide, is one of the oldest thiazide diuretics. Recently, CAN has been difficult to be separated from HCT in most tablets [1, 2]. Candesartan is an effective, irreversible antagonist because it has the highest known receptor affinity of all the ARBs, and high doses of angiotensin II (Ang II) don't push it off the receptor. Study after study has shown the positive effects of candesartan cilexetil in the treatment of high blood pressure and heart failure (HF) [3]. For more than 50 years, thiazide-type diuretic hydrochlorothiazide (HCT) has been available for clinical usage [4]. HCT is also used to lower blood pressure while walking, which is mostly caused by a drop in blood pressure at night [5]. In clinical trials lasting anywhere from 8 weeks to 3 years, the fixed-dose combination medication candesartan and hydrochlorothiazide has emerged as a key option in the treatment of hypertension due to its great efficacy in lowering blood pressure and preventing damage to target organs [6].

Various mathematical techniques have been developed for the use of chemometric strategies, such as partial least squares, so it is possible to study drug –excipient interactions in a single sample without resorting to costly and time-consuming chemical separation [7], and multiple linear regression [8, 9], The *H*-point standard addition method (HPSAM) is also used to assess binary mixtures in chemometric techniques [10]. It was afterward changed to multicomponent analysis [11–13]. The *Q*-absorption ratio method [14, 15], and the correction absorbance method have also been used for binary mixture analysis [16, 17].

Several analytical methods have been reported for the estimation of these ingredients individually or simultaneously, individual measurements of CAN and HCT by different analytical methods appeared in a few reported works, for an instant, valsartan look alike candesartan cilexetil as by high-performance liquid chromatography [18–20], gas chromatography–mass spectroscopy, and liquid chromatography [21, 22]. Also, hydrochlorothiazide is determined by liquid chromatography [23, 24], capillary zone electrophoresis [25], and spectrophotometry [26, 27]. The combined dosage of CAN and HCT was resolved simultaneously by different analytical methods like high-performance liquid chromatography [28, 29], high-performance thin layer chromatography [30], liquid chromatography, mass spectroscopy [31, 32], liquid chromatography [33, 34], derivative spectrophotometry [5], and the derivative ratio method [35].

**Figure 1:**
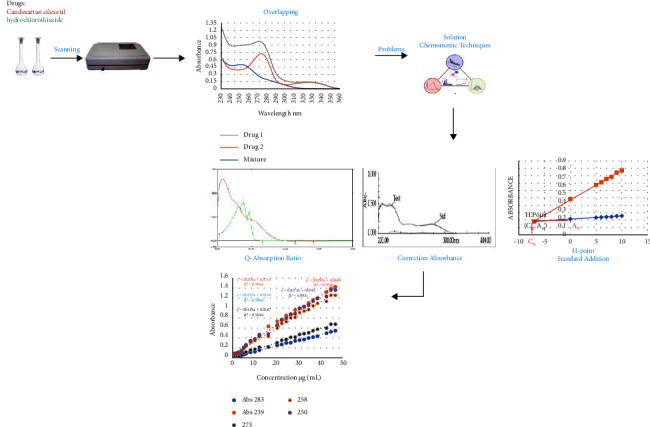
Schematic diagram of the proposed methods.

In this work, HPSAM, the *Q*-absorbance ratio technique, and the correction absorbance method were used for the simultaneous assessment of CAN and HCT. These processes were accurate, selective, sensitive, and reasonably priced. These new techniques eliminate strongly overlapped spectra. The outcomes were contrasted with those attained using the HPLC technique. A simple graphical description of the suggested method is depicted in Figure 1.

## 2. Theoretical Background

### 2.1. *H*-Point Standard Addition Method

**Figure 2:**
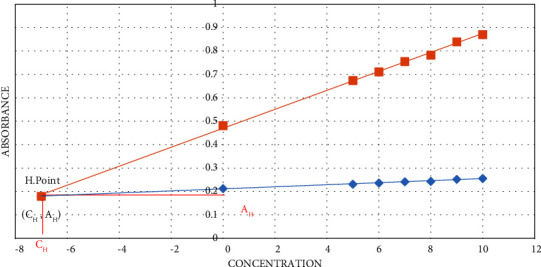
*H*-point standard addition method.

This technique plots the analytical signal versus the amount of analyte at two wavelengths. Considering a sample interference, *Y* as the interference and *X* as the analyte. For HPSAM to determine *X* concentration, interference absorbance must be constant [10]. Two straight lines with a common point in *H* (−*C*_*H*_, *A*_*H*_) are depicted in Figure 2 [13].

The measured quantity of *X* is added. Finally, absorbance at two specified wavelengths is measured according to the following equations:

(1) $$\begin{array}{c} A_{\left(\lambda 1\right)}=b_{0}+b+M_{\lambda 1}C_{i\;}\text{,} \end{array}$$

(2) $$\begin{array}{c} A_{\left(\lambda 2\right)}=A_{0}+A^{`}+M_{\lambda 2}C_{i}\text{,} \end{array}$$

where *A*_(*λ*1)_ and *A*_(*λ*2)_ represent the absorbances at *λ*_1_ and *λ*_2,_ respectively, *b*_0_ and *A*_0_ represent the analytical signals of *X* at *A*_*λ*1_ and *A*_*λ*2_ (*b*_0_ ≠ *A*_0_), and *b* and *A*^`^ represent the analytical signals of *Y* at *A*_*λ*1_ and *A*_*λ*2_ (*b* = *A*^`^). *M*_*λ*1_ and *M*_*λ*2_ are the slopes of the calibration lines at *λ*_1_ and *λ*_2_. Lastly, *C*_i_ signifies the addition of *X*. As illustrated in Figure 2, the *H*-point is dependent on the analyte concentration.

Since *C*_*i*_ = *C*_*H*_ is derived from equations (1) and (2), where *A*_*λ*1_ = *A*_*λ*2_

(3) $$\begin{array}{c} b_{0}+b+M_{\lambda 1}\left(-C_{H}\right)=A_{0}+A^{`}+M_{\lambda 2}\left(-C_{H}\right)\text{.} \end{array}$$

Hence,

(4) $$\begin{array}{c} {-C}_{H}=\frac{\left[\left(A_{0}-b_{0}\right)+\left(A^{`}-b\right)\right]}{M_{\lambda 1}-M_{\lambda 2}}\text{.} \end{array}$$

As interference *Y* has identical absorbance values at *λ*_1_ and *λ*_2,_*A*^`^ = *b* and

(5) $$\begin{array}{c} -C_{H}=\frac{\left(A_{0}-b_{0}\right)}{M_{\lambda 1}-M_{\lambda 2}}\text{.} \end{array}$$

Which fits within the given equation.

(6) $$\begin{array}{c} -C_{H}=-\frac{b_{0}}{M_{\lambda 1}}=-\frac{A_{0}}{M_{\lambda 2}}\text{.} \end{array}$$

That −*C*_*H*_ is proportional to the amount of analyte present in the mixture can be concluded [36].

*A*_*H*_, the intersection point's ordinate value can be expressed as follows:

(7) $$\begin{array}{c} A_{H}=b_{0}+b+M_{\lambda 1}\left(-C_{H}\right)\text{.} \end{array}$$

As *b*_0_ = *M*_*λ*1_ from equation (7), then *A*_*H*_ = *b* and *A*_*H*_ = *A*^`^.

The relationship between absorbance at specific wavelengths and the *H*-point (*A*_*H*_) is solely due to interference. Since this is the same as the zero point on the calibration graph for the analyte when the sample is present, the analytical signal can be used to figure out how much *Y* there is from the calibration graph.

The following guidelines were used to choose the best wavelength combination for the determination of CAN and HCT in a binary mixture by HPSAM:

1. At certain wavelengths, analyte signals must be linear to concentration, and the interferent signal must be unaffected by analyte concentration
2. The analytical signal from a mixture combining analyte and interferent should equal the sum of their individual signals
3. For reasonable sensitivity and accuracy, the slope difference between two straight lines measured at *λ*_1_ and *λ*_2_ must be as large as feasible [37, 38]

### 2.2. *Q*-Absorption Ratio Method

This approach is applicable to medications that follow Beer's law at all wavelengths and have a consistent ratio of absorbance between any two wavelengths [39]. This method utilizes the ratio of absorption at two chosen wavelengths. One represents the drug's maximal absorbance, while the other represents the iso-absorptive point. Assume that *X* and *Y* are the two medications.

The following equations were combined depending on this relationship: *ax*_1_ = *ay*_1_ at *λ*_1_ and *L* = 1.

(8) $$\begin{array}{c} At\;{\;\lambda }_{1\;}\;A_{1}={ax}_{1}C_{X}+{ax}_{1}C_{y}\;\left(because\;{ax}_{1}={ay}_{1}\right)\text{,} \end{array}$$

At

(9) $$\begin{array}{c} \lambda_{2\;}{\;A}_{2}={ax}_{2}C_{X}+{ay}_{2}C_{y}\text{.} \end{array}$$

Equation (9) divided by equation (8), we get

(10) $$\begin{array}{c} \frac{A_{2}}{A_{1}}=\frac{{ax}_{2}C_{X}+{ay}_{2}C_{y}}{{ax}_{1}C_{X}+{ax}_{1}C_{y}}\text{.} \end{array}$$

When *F*_*x*_=*C*_*X*_/*C*_*X*_+*C*_*y*_, *F*_*y* _=*C*_*y*_/*C*_*X*_+*C*_*y*_

(11) $$\begin{array}{c} \frac{A_{2}}{A_{1}}=\frac{{ax}_{2}F_{X}+\left({ay}_{2}-{ay}_{2}F_{x}\right)}{{ax}_{1}}\text{.} \end{array}$$

*A*_2_/*A*_1_=*ax*_2_*F*_*X*_/*ax*_1_ − *ay*_2_*F*_*y*_/*ay*_1_+*ay*_2_/*ay*_1_because(*ax*_1_=*ay*_1_)

Let *Q*_*X*_=*ax*_2_/*ax*_1_, *Q*_*Y*_=*ay*_2_/*ay*_1_, *Q*_*M*_=*A*_2_/*A*_1_

So *Q*_*M*_=*F*_*X*_*Q*_*X*_ − *F*_*y*_*Q*_*Y*_+*Q*_*Y*_

(12) $$\begin{array}{c} F_{x}=\frac{Q_{M}-Q_{Y}}{Q_{X}-Q_{Y}}\text{.} \end{array}$$

Equation (12), which is approximate rather than exact, yields the percentage rather than the concentration of *X* and *Y* in the mixture.

If we rearrange equation (8) to include the absolute concentrations of *X* and *Y*, we obtain the following equation:

(13) $$\begin{array}{c} C_{x}+C_{y}=\frac{A_{1}}{{ax}_{1}}\text{.} \end{array}$$

From equations (12) and (13), we get

(14) $$\begin{array}{c} \frac{C_{x}}{A_{1}/{ax}_{1}}=\frac{Q_{M}-Q_{Y}}{Q_{X}-Q_{Y}}\text{,} \end{array}$$

(15) $$\begin{array}{c} C_{x}=\frac{Q_{M}-Q_{Y}}{Q_{X}-Q_{Y}}*\frac{A_{1}}{{ax}_{1}}\text{,} \end{array}$$

(16) $$\begin{array}{c} C_{y}=\frac{Q_{M}-Q_{Y}}{Q_{X}-Q_{Y}}*\frac{A_{2}}{{ay}_{1}}\text{,} \end{array}$$

where *C*_*x*_ and *C*_*y*_ are the *X* and *Y* concentrations, respectively, *A*_1_, *A*_2_ are the absorbances of the mixture at *λ*_1_, *λ*_2_, *ax*_1_, and *ay*_1_ are absorptivities of *X* and *Y* at 261 nm, and *ax*_2_ and *ay*_2_ are absorptivities of *X* and *Y* at 270 nm.

Equations (15) and (16) give the absolute concentration values of drug *X* and *Y* [15, 40, 41].

### 2.3. Correction Absorbance Method

In this method, *λ*_max_ of analyte and interference was determined by scanning the drug solution in the UV Spectrophotometer. Which requires two wavelengths, one is the *λ*_max_ of the analyte and the second one is the wavelength in which the analyte has no absorbance, the signal is only related to interference; thus, the absorbance of the interference at the first wavelength was calculated as follows [42]:

(17) $$\begin{array}{c} A_{corr\;\lambda 1}=A_{mix\;\lambda 1\;}-\left(r_{1}\times A_{mix\;\lambda 2}\right)\text{.} \end{array}$$

*A*_mix*λ*1_, and *A*_mix*λ*2_, are the absorbance of the mixture at *λ*_1_, and *λ*_2_. *A*_corr*λ*1_ are the net absorbances at *λ*_1_ nm, The slope ratios of the interference calibration graph are represented by the values *r*_1_ and *r*_2_, respectively [16, 43].

(18) $$\begin{array}{c} r_{1}=\frac{{Slope}_{ƛ1}}{{Slope}_{ƛ}}\text{.} \end{array}$$

## 3. Material and Methods

### 3.1. Apparatus

The UV-visible spectrophotometer (UV-VIS/VIS spectrophotometer AE-S60) was connected to an identical 1.0 cm quartz cell for the UV-VIS scanning spectrum.

All of the measurements in this study were estimated with the MetaSpec Pro software suite.

### 3.2. Preparation of Real Sample

The average mass of 10 pills was measured, they were mashed, the powder was added to a 1 : 1 NaOH : ethanol solution, and it was continuously stirred for 10 minutes. The next step is the filtering procedure. Three times, 10 ml of 1 : 1 NaOH : ethanol was used to wash the powder off the filter paper. After that, the solution was finished to a final volume of 1 L of 1 : 1 NaOH : ethanol. The solution was stored in a 4°C refrigerator.

### 3.3. Preparation of Standard Solution

Preparing a 1000 *µ*g/mL HCT solution by dissolving 0.025 gm HCT in 1 : 1 NaOH : ethanol, to attain the needed analyte concentration, was diluted in a 25 mL volumetric flask. A 1000 *µ*g/mL CAN solution was made by dissolving 0.025 gm CAN in 1 : 1 NaOH : ethanol and was diluted in a 25 mL volumetric flask. These solutions were stored at 4°C in darkness. By serially diluting solutions with 1 : 1 NaOH : ethanol, more diluted solutions were prepared. These solutions were stored at 4°C in darkness. By serially diluting solutions with 1 : 1 NaOH : ethanol, more diluted solutions were prepared.

### 3.4. 1 : 1 NaOH : Ethanol Preparation

0.2 N NaOH was prepared by dissolving 4 gm of NaOH in deionized water and was diluted in a 500 mL volumetric flask. Then mixed with ethanol one by one to make the solvent mixture 1 : 1 NaOH : ethanol.

## 4. Procedures

### 4.1. *H*-Point Standard Addition Method

**Figure 3:**
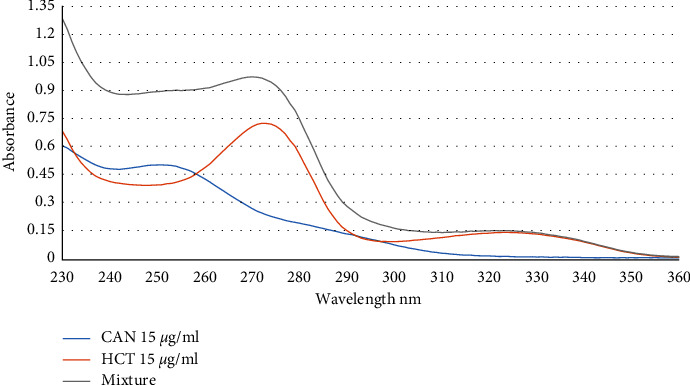
Absorption spectra of 15 *µ*g/ml candesartan cilexetil and 15 *µ*g/ml hydrochlorothiazide.

Following is the general approach for determining CAN and HCT in a binary combination. An aliquot of a solution containing 15 *µ*g/mL CAN, and 15 *µ*g/mL HCT was added to a 2 mL volumetric flask, which was then filled to the mark with deionized water. The solution was then allowed to stand for five minutes at room temperature. The absorbance of the solution at the specified wavelengths was then measured by transferring a portion of the solution into a quartz cell. Standard additions of CAN ranging from 3 to 13 *µ*g/mL were done on the synthetic sample, which included a variable ratio of CAN to HCT. Simultaneous determination of CAN and HCT was conducted using HPSAM at two selected wavelengths. The wavelengths selected depend on the principle of HPSAM as mentioned above, as well as the absorbance for the analyte was different and constant for interference at selected wavelengths of 239 and 283 nm, as shown in Figure 3, where *C*_*H*_ is the unknown analyte concentration of CAN, and *A*_*H*_ is the analytical signal of interference HCT, was determined at 283 nm in the calibration curve of standard HCT with *y* = 0.0247*x* + 0.0297 regression equation and 0.9985 correlation coefficient.

### 4.2. *Q*-Absorption Ratio Method

The CAN and HCT in a binary mixture were determined by the following procedure. The mixtures of standard solutions of the drugs were prepared with a 2 mL volumetric flask, in different concentration ratios in the range of 11–19 *µ*g/mL by diluting the appropriate volume of a stock solution of each drug with deionized water, then the solution was transferred to a quartz cell to scan in the range of 200–400 nm. The determination is carried out by *Q*-absorption ratios at two selected wavelengths. The selection of wavelengths was carried out related to the principle of the *Q*-Absorption ratio method, where one of these wavelengths is the iso-absorptive point and the other one is the max of one of the drugs. After different wavelengths were tested, 273 nm was selected as *λ*_max_ of HCT and 258 nm as the iso-absorptive point of CAN and HCT for applying the *Q*-absorption ratio procedure, as shown in Figure 3. HCT and CAN were determined at 273 and 258 nm with a *Q*-absorption ratio in the following equations:

(19) $$\begin{array}{c} C_{x}=\frac{Q_{M}-Q_{Y}}{Q_{X}-Q_{Y}}*\frac{A_{1}}{{ax}_{1}}\text{,}\\ C_{y}=\frac{Q_{M}-Q_{Y}}{Q_{X}-Q_{Y}}*\frac{A_{2}}{{ay}_{1}}\text{,} \end{array}$$

where *C*_*x*_ and *C*_*y*_ are the HCT, and CAN concentrations, respectively, *A*_1_ *andA*_2_ are the absorbances of the mixture at *λ*_1_*and* *λ*_2_, *ax*_1_, and *ay*_1_ are absorptivities of HCT and CAN at 273 nm, and *ax*_2_ and *ay*_2_ are absorptivities of HCT and CAN at 258 nm.

### 4.3. Correction Absorbance Method

The following procedure was applied for the determination of HCT and CAN with the correction absorbance method. The series standard solution was prepared by transferring the aliquot amounts of stock solution to a 2 mL volumetric flask and completed to the mark with deionized water. The solution was then poured into the 1 cm quartz cell and scanned in the range of 200–400 nm. HCT, and CAN were determined by the correction absorbance method when the selected pair of wavelengths returned to the principle of the method as explained above. The first wavelength is 250 nm *λ*_max_ of CAN, and the second one is 340 nm for direct determination of HCT and applying the correction absorbance equation for the removal of the absorbance of HCT at 250 nm. Finally, CAN was determined at the calibration curve of standard CAN with *y* = 0.0295*x* + 0.0648 regression equation and 0.9926 correlation coefficient, and HCT was determined at the calibration curve of standard HCT with *y* = 0.0054*x* + 0.0067 regression equation and 0.9979 correlation coefficient.

## 5. Linear Range

**Figure 4:**
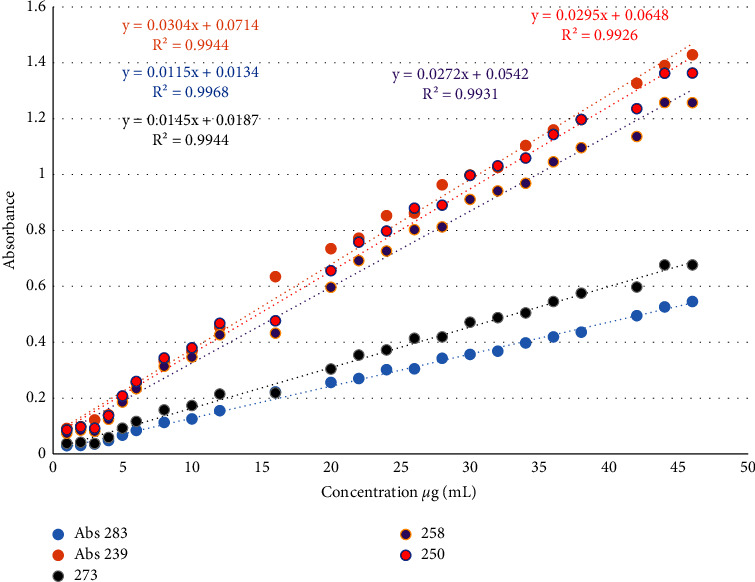
Calibration graph of candesartan cilexetil at 283, 273, 258, 250, and 239 nm.

**Figure 5:**
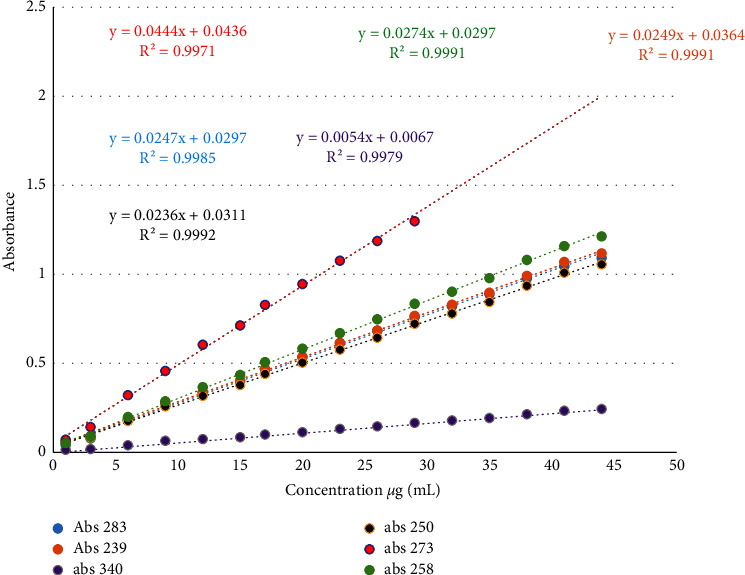
Calibration graph of hydrochlorothiazide at 340, 283, 273, 258, 250, and 239 nm.

The calibration curve was drawn for selected wavelengths related to the procedures of the techniques, namely, 239 and 283 nm for the HPSAM, 273 and 258 nm for the Q-absorption ratio method, and 250 and 340 nm for the correction absorbance method. As shown in Figures 4 and 5.  Table 1 shows the linear range of drugs for all methods at all wavelengths.

## 6. Limit of Detection (LOD) and Limit of Quantification (LOQ)

### 6.1. *H*-Point Standard Addition Method and Correction Absorbance Method

Equations (20) and (21) provide the computations for the limit of detection (LOD) and limit of quantification (LOQ) for the *H*-point standard addition method and correction absorbance method.

(20) $$\begin{array}{c} LOD=X_{b}+3SD_{b}\text{,} \end{array}$$

(21) $$\begin{array}{c} LOQ=X_{b}+10SD_{b}\text{,} \end{array}$$

where *X*_*b*_ represents the concentration of five replications (*n* = 5) and SD_*b*_ is the standard deviation of the blank [13]. The corresponding values obtained for HCT were 0.46 *µ*g/mL LOD and 0.91 *µ*g/mL LOQ, and for CAN, they were 0.48 *µ*g/mL LOD and 1.26 *µ*g/mL LOQ in HPSAM as shown in Tables 2 and 3, and 0.93 *µ*g/mL LOD and 2.1 *µ*g/mL LOQ for HCT and 0.94 *µ*g/mL LOD and 2.4 *µ*g/mL LOQ for CAN in the correction absorbance method as shown in Tables 4 and 5.

### 6.2. *Q*-Absorption Ratio Method

Calibration curves were used to figure out the LOD and LOQ of the new method by the following equation:

(22) $$\begin{array}{c} LOD=\frac{3.3\;\sigma }{S}\text{,}\\ LOQ=\frac{10\;\sigma }{S}\text{,} \end{array}$$

where *σ* is the standard deviation of the blank and *S* is the slope of the calibration curve.  Table 6 shows the LOD and LOQ for those drugs [14].

## 7. Accuracy and Precision

By using the methods for assessing various ratios of the drug combination, the suggested methods' accuracy was evaluated, by preparing the following combinations for CAN and HCT, respectively (15 : 11, 15 : 13, 15 : 15, 17 : 15, and 19 : 15) *µ*g/mL. Then, using the relevant regression equation, all the suggested techniques were used to obtain the desired concentration. For the *H*-point standard addition method, *Q*-absorption ratio method, and correction absorbance technique, respectively, accuracy was expressed as a percentage error, which was displayed in Tables 7–9.

Additionally, the accuracy of the suggested procedures was examined by measuring the drug concentrations in a 15 g/mL combination five times in a row. For the *H*-point standard addition method, *Q*-absorption ratio method, and correction absorbance technique, respectively, the accuracy of each approach is shown as a percentage of the relative standard deviation in Tables 10–12.

## 8. Interferences

The tolerance limit was described as the concentration of the added species interference (such as lactose monohydrate, magnesium stearate, stearic acid, polyethylene glycol, starch, sucrose, Na_2_CO_3_, and NaHCO_3_) causing an error of more than ±5% on the analytical signal, and then, before the beginning of the process with the analysis of the compound under study in pharmaceutical dosage forms, it was conducted to discover its effect. Samples were prepared by mixing known quantities of the investigated drugs with different quantities of mutual excipients. The result shows magnesium stearate, stearic acid, and Na_2_CO_3_ were insoluble in 1 : 1 NaOH : ethanol, also, the result of the methods in the determination of drug in the presence of soluble interferences shows a good percentage recovered shows that there is no interference from these supplement additives with the methods applied. The results obtained in Tables 13–15 reveal a great degree of accuracy for all methods.

## 9. Application

These procedures and methods have been used in pharmaceutical formulations (tabs) and synthetic lab mixtures to assess the analytical applicability of the intended methodologies. These methods are frequently used for simultaneous determination. All of our methods' results were contrasted with the HPLC result, which served as the benchmark. The HPSAM was used for the simultaneous estimation of HCT and CAN in the synthetic mixture and pharmaceutical formulation. The results are listed in Table 16. The *Q*-analysis technique procedure was effectively used to determine the amounts of HCT and CAN by being repeated three times within the synthetic lab mixture and pharmaceutical formulation, as shown in.

Table 17, the results of the correction absorbance technique for simultaneous determination of HCT and CAN in the pharmaceutical formulation, are shown in Table 18. The value of the real samples was calculated for each of the tablets by the HPLC method. According to the tables, the methods presented in this work are sufficiently general to be applied to figure out the HCT and CAN of a real sample of tablets simultaneously.

## 10. Results and Discussion

Based on the results, we made the following observations. Experimental evaluation of the HPSAM, *Q*-absorption ratio, and correction absorption methods in this work led us to consider these methods effective for the simultaneous determination of HCT and CAN. Our results that were presented in this work are generally sufficient to be applied to real samples in pharmaceutical formulations. The effectiveness of the proposed methods has been substantiated in Table 19. The spectra of the binary mixture that was prepared in accordance with Section 3.3 are shown in Figure 1. As can be seen, the samples' analytes and interference spectra exhibit significant wavelength range overlap. Following the testing of numerous wavelength pairings for the use of HPSAM, *Q*-absorption ratio, and correction absorbance methods, HCT and CAN function in this technique as analyte and interference. The findings indicate that 239 and 283 nm are best for determining CAN and HCT by HPSAM, while 273 and 258 nm are best for the *Q*-absorption ratio, finally, 250 and 340 nm were chosen for the correction absorbance method, because there is no interference at these wavelengths. In light of this, we proposed new methods: HPSAM, *Q*-absorption ratio, and absorbance correction to simultaneously determine HCT and CAN. We can come up with some hypotheses regarding the reproducibility of the procedure based on the outcomes of the five separate measurements. The proposed methods were validated according to the ICH recommendations [44]. These methods were utilized successfully to estimate the quantities of candesartan, cilexetil, and hydrochlorothiazide in commercially available tablet formulations containing candesartan cilexetil and hydrochlorothiazide. Three tablet formulations were used as samples in this study, one of these samples is Candex which contains in its composition 12.07 mg per tablet of HCT and 15.89 mg per tablet of CAN as analyzed by the standard method using HPLC. Using the *H*-point standard addition method, the amount of HCT was found to be 12.08 mg and the amount of CAN was found to be 15.79 mg, which correspond to 99.917 percent and 100.64 percent of the w/w label claim, respectively. Using the *Q*-absorption ratio method, the amount of HCT was found to be 12.1 mg and the amount of CAN was found to be 15.9 mg, which corresponds to 100.2 percent and 100.06, respectively. The last method used in this study is the correction absorbance method, and the amount of HCT found in the tablet formulation was 12.56 mg for HCT and 16.21 mg for CAN, which corresponds to 104.1 and 102 percent, respectively. For all medicines, recovery and error percentages were used to calculate accuracy. The data comparison between our methods and the standard HPLC method is shown in Tables 16–18.

## 11. Conclusion

A brand-new, straightforward, quick, and sensitive approach is suggested for the analysis of two binary mixtures with overlapping spectra. The process starts with the creation of absorbance ratio spectra, then moves on to measuring peak-to-trough amplitudes. The suggested methods have various advantages over conventional spectrophotometric methods for the resolution of binary mixtures, including the lack of a need for complex mathematical handling of the absorption data. In an ongoing study, straightforward and effective chemometric methods like *H*-point standard addition, *q*-absorption ratio, and correction absorbance methods were devised for the simultaneous measurement of hydrochlorothiazide and candesartan in bulk and in the pharmaceutical dosage form. It was found that the validity of these methods could be demonstrated through the accurate and precise determination of drug combinations in a variety of laboratory-prepared mixtures and pharmaceutical tablets. Consequently, the methodology proposed here is suitable for routine quality control of these set mixtures.

## Figures and Tables

**Table 1:** Linearity of drugs at all proposed methods.

Method	Wavelength (nm)	Candesartan cilexetillinearity (*µ*g/mL)	Hydrochlorothiazide linearity (*µ*g/mL)
HPSAM	239	1–46	1–44
	283	1–46	1–44

*Q*-absorption ratio method	273	1–46	1–29
	258	1–46	1–44

Correction absorbance method	250	1–46	1–44
	340	1–46	

**Table 2:** Limit of detection (LOD) and limit of quantification (LOQ) of HCT by HPSAM.

Λ	Regression equation	*R* ^2^	Added (*µ*g/mL)		Found (*µ*g/mL)	
			HCT	CAN	HCT	CAN
283	*Y* = 0.01158^*∗*^*X* + 0.1783	0.9915	15	15	0.21	14.9
239	*Y* = 0.03315^*∗*^*X* + 0.4996	0.9955				

283	*Y* = 0.01152^*∗*^*X* + 0.1767	0.9905	15	15	0.2	14.85
239	*Y* = 0.03315^*∗*^*X* + 0.4978	0.9955				

283	*Y* = 0.01147^*∗*^*X* + 0.1813	0.9911	15	15	0.33	15.02
239	*Y* = 0.03295^*∗*^*X* + 0.5039	0.9962				

283	*Y* = 0.01141^*∗*^*X* + 0.1787	0.9917	15	15	0.23	15.12
239	*Y* = 0.03276^*∗*^*X* + 0.5014	0.9968				

283	*Y* = 0.01136^*∗*^*X* + 0.1800	0.9922	15	15	0.33	15.05
239	*Y* = 0.03282^*∗*^*X* + 0.5029	0.9967				

Mean					0.26	
SD					0.065	
LOD					0.46	
LOQ					0.91	

^*∗*^^1^ calculated using the regression equation of *Y* = 0.0247*X* + 0.0297 and the HCT calibration curve at 283 nm.

**Table 3:** Limit of detection (LOD) and limit of quantification (LOQ) of CAN by HPSAM.

*λ*	Regression equation	*R* ^2^	Added (*µ*g/mL)		Found (*µ*g/mL)	
			HCT	CAN	HCT	CAN
283	*Y* = 0.01152^*∗*^*X* + 0.4192	0.9905	15	15	15.2	0.07
239	*Y* = 0.03433^*∗*^*X* + 0.4208	0.9962				

283	*Y* = 0.01103^*∗*^*X* + 0.4202	0.9944	15	15	15.13	0.33
239	*Y* = 0.03432^*∗*^*X* + 0.4278	0.9925				

283	*Y* = 0.01245^*∗*^*X* + 0.4128	0.9904	15	15	15.02	0.05
239	*Y* = 0.03637^*∗*^*X* + 0.4116	0.9945				

283	*Y* = 0.01103^*∗*^*X* + 0.4154	0.9944	15	15	15.03	0.14
239	*Y* = 0.03386^*∗*^*X* + 0.4185	0.9922				

283	*Y* = 0.01141^*∗*^*X* + 0.4182	0.9917	15	15	15.25	0.16
239	*Y* = 0.03510^*∗*^*X* + 0.4144	0.9977				

Mean						0.15
SD						0.111
LOD						0.48
LOQ						1.26

^*∗*^^1^ calculated using the regression equation of *Y* = 0.0054*x* + 0.0067 and the HCT calibration curve at 340 nm. ^*∗*^^2^ calculated using the regression equation of *Y* = 0.0295*x* + 0.0648 and the HCT calibration curve at 250 nm.

**Table 4:** Limit of detection (LOD) and limit of quantification (LOQ) of HCT by correction absorbance.

*λ*	Added (*µ*g/mL)		Found (*µ*g/mL)	
	HCT	CAN	HCT	CAN
340	0	15	0.65	14.67
250				

340	0	15	0.23	14.8
250				

340	0	15	0.56	14.91
250				

340	0	15	0.45	14.89
250				

340	0	15	0.38	14.8
250				

Mean			0.45	
SD			0.1623	
LOD			0.93	
LOQ			2.1	

^*∗*^^1^ calculated using the regression equation of *Y* = 0.0236*x* + 0.0311 and the HCT calibration curve at 283 nm.

**Table 5:** Limit of detection (LOD) and limit of quantification (LOQ) of CAN by correction absorbance.

*λ*	Added (*µ*g/mL)		Found (*µ*g/mL)	
	HCT	CAN	HCT	CAN
340	15	0	14.61	0.54
250				

340	15	0	1482	0.45
250				

340	15	0	14.96	0.036
250				

340	15	0	14.88	0.51
250				

340	15	0	14.93	0.39
250				

Mean				0.39
SD				0.2035
LOD				0.94
LOQ				2.4

^*∗*^^1^ calculated using the regression equation of *Y* = 0.0236*x* + 0.0311, and the HCT calibration curve at 283 nm.

**Table 6:** LOD and LOQ for HCT and CAN by the *Q*-absorbance ratio method.

Parameter	Hydrochlorothiazide	Candesartan cilexetil
Determination wavelength	273 nm *λ*_max_	258 nm isoabsorptive point
LOD (*µ*g/mL)	0.76	0.88
LOQ (*µ*g/mL)	1.93	2.1

**Table 7:** Accuracy of the *H*-point standard addition method in the determination of HCT and CAN.

*λ*	Regression equation	*R* ^2^	Hydrochlorothiazide (HCT)^*∗*^^1^ (*µ*g/mL)			Candesartan cilexetil (CAN) (*µ*g/mL)		
			Add	Found	%*E*	Add	Found	%*E*
283	*Y* = 0.01141^*∗*^*X* + 0.5420	0.9917	15	15.3	1.7	11	10.7	2.9
239	*Y* = 0.03298^*∗*^*X* + 0.7723	0.9961						

283	*Y* = 0.01147^*∗*^*X* + 0.5494	0.9911	15	14.6	2.8	13	12.9	0.7
239	*Y* = 0.03315^*∗*^*X* + 0.8294	0.9955						

283	*Y* = 0.01141^*∗*^*X* + 0.5917	0.9917	15	15.4	2.5	15	14.7	1.8
239	*Y* = 0.03309^*∗*^*X* + 0.9109	0.9957						

283	*Y* = 0.01154^*∗*^*X* + 0.6523	0.9903	17	16.97	0.18	15	15.45	3
239	*Y* = 0.03327^*∗*^*X* + 0.9882	0.9950						

283	*Y* = 0.01155^*∗*^*X* + 0.6745	0.9902	19	19.1	0.5	15	15.14	0.9
239	*Y* = 0.03312^*∗*^*X* + 1.001	0.9956						

^*∗*^^1^ calculated using the regression equation of *Y* = 0.0247*x* + 0.0297, and the HCT calibration curve at 283 nm.

**Table 8:** Accuracy of the *Q*-absorption ratio method in the determination of HCT and CAN.

*λ*	Hydrochlorothiazide (*µ*g/mL)			Candesartan cilexetil (*µ*g/mL)		
	Add	Found	%*E*	Add	Found	%*E*
273	15	14.8	−1.3	11	11.3	2.73
258						

273	15	14.57	−2.76	13	12.48	−3.98
258						

273	15	15.02	0.13	15	15.03	0.2
258						

273	17	16.9	−0.59	15	14.96	−0.27
258						

273	19	18.77	−1.2	15	14.85	−1
258						

**Table 9:** Accuracy of the correction absorbance method in the determination of HCT and CAN.

*λ*	Hydrochlorothiazide (*µ*g/mL) ^*∗*^			Candesartan cilexetil (*µ*g/mL)		
	Add	Found	%*E*	Add	Found	%*E*
340	15	15.12	0.8	11	11.01	0.1
250						

340	15	14.45	−3.66	13	12.7	−2.29
250						

340	15	15.31	2.06	15	14.86	−0.95
250						

340	17	16.54	−2.73	15	14.6	2.6
250						

340	19	19.51	2.67	15	14.87	−0.9
250						

^*∗*^^1^ calculated using the regression equation of *Y* = 0.0054*x* + 0.0067 and the HCT calibration curve at 340 nm. ^*∗*^^2^ calculated using the regression equation of *Y* = 0.0295*x* + 0.0648 and the HCT calibration curve at 250 nm.

**Table 10:** Precision of the *H*-point standard addition method in the determination of HCT and CAN.

*λ*	Regression equation	*R* ^2^	Added (*µ*g/mL)		Found (*µ*g/mL)	
			HCT	CAN	HCT	CAN
283	*Y* = 0.01103^*∗*^*X* + 0.5816	0.9944	15	15	15.12	14.98
239	*Y* = 0.03188^*∗*^*X* + 0.8939	0.9989				

283	*Y* = 0.01096^*∗*^*X* + 0.5688	0.9945	15	15	14.75	14.86
239	*Y* = 0.03194^*∗*^*X* + 0.8805	0.9985				

283	*Y* = 0.01141^*∗*^*X* + 0.5638	0.9917	15	15	14.43	14.59
239	*Y* = 0.03260^*∗*^*X* + 0.8730	0.9974				

283	*Y* = 0.01108^*∗*^*X* + 0.5719	0.9941	15	15	14.71	15.06
239	*Y* = 0.03187^*∗*^*X* + 0.8850	0.9989				

283	*Y* = 0.01103^*∗*^*X* + 0.5793	0.9944	15	15	15.17	14.64
239	*Y* = 0.03186^*∗*^*X* + 0.8843	0.9989				

Mean					14.84	14.83
SD					0.3084	0.2061
RSD_(*n*_ _=_ _5)_					2.08	1.39
%R					98.9	98.84

^*∗*^^1^ calculated using the regression equation of *Y* = 0.0247*x* + 0.0297 and the HCT calibration curve at 283 nm.

**Table 11:** Precision of the *Q*-absorption ratio method in the determination of HCT and CAN.

*λ*	Added (*µ*g/mL)		Found (*µ*g/mL)	
	HCT	CAN	HCT	CAN
273	15	15	14.78	14.84
258				

273	15	15	15	15
258				

273	15	15	15	15
258				

273	15	15	14.6	15.1
258				

273	15	15	15	15
258				

Mean			14.88	14.99
SD			0.1813	0.09338
RSD_(*n*_ _=_ _5)_			1.22	0.63
%*R*			99.2	99.93

**Table 12:** Precision of the correction absorbance method in the determination of HCT and CAN.

*λ*	Added (*µ*g/mL)		Found (*µ*g/mL)	
	HCT	CAN	HCT	CAN
340	15	15	14.72	14.71
250				

340	15	15	15.22	15.23
250				

340	15	15	15.13	14.93
250				

340	15	15	15.27	15.14
250				

340	15	15	15.2	15.24
250				

Mean			15.11	15.05
SD			0.2226	0.2273
RSD_(*n*_ _=_ _5)_			1.47	1.51
%*R*			100.73	100.33

^*∗*^^1^ calculated using the regression equation of *Y* = 0.0054*x* + 0.0067 and the HCT calibration curve at 340 nm. ^*∗*^^2^ calculated using the regression equation of *Y* = 0.0295*x* + 0.0648 and the HCT calibration curve at 250 nm.

**Table 13:** Effect of interferences on the *H*-point standard addition method.

*λ*	Regression equation	*R* ^2^	Type of interferences	Amount of interferences (*µ*g/mL)	HCT (*µ*g/mL)			CAN (*µ*g/mL)			
					Add	Found	%*E*	Add	Found		%*E*
283	*Y* = 0.01202^*∗*^*X* + 0.5936	0.9946	Polyethylene glycol	100	15	14.98	0.15	15		15.08	0.51
239	*Y* = 0.03424^*∗*^*X* + 0.9286	0.9901									

283	*Y* = 0.01219^*∗*^*X* + 0.6102	0.9923	Sucrose	100	15	15.6	4.07	15		14.8	−1.35
239	*Y* = 0.03424^*∗*^*X* + 0.9365	0.9901									

283	*Y* = 0.01155^*∗*^*X* + 0.6039	0.9902	Lactose	100	15	15.5	3.4	15		15.3	2
239	*Y* = 0.03353^*∗*^*X* + 0.9402	0.9938									

283	*Y* = 0.01165^*∗*^*X* + 0.5931	0.9965	Starch	100	15	14.96	0.29	15		15.6	3.74
239	*Y* = 0.03449^*∗*^*X* + 0.9485	0.9995									

283	*Y* = 0.01115^*∗*^*X* + 0.5776	0.9974	NaHCO_3_	100	15	14.95	0.34	15		14.89	−0.74
239	*Y* = 0.03320^*∗*^*X* + 0.9059	0.9993									

283	*Y* = 0.01197^*∗*^*X* + 0.6095	0.9967	All of above	100	15	15.5	3.4	15		15.23	1.54
239	*Y* = 0.03407^*∗*^*X* + 0.9461	0.9996									

^*∗*^^1^ calculated using the regression equation of *Y* = 0.0247*x* + 0.0297 and the HCT calibration curve at 283 nm.

**Table 14:** Effect of interferences on the *Q*-absorption ratio method.

*λ*	Type of interferences	Amount of interferences (*µ*g/mL)	HCT (*µ*g/mL)			CAN (*µ*g/mL)		
			Add	Found	%*E*	Add	Found	%*E*
273	Polyethylene glycol	100	15	15.36	2.4	15	15.41	2.7
258								

273	Sucrose	100	15	14.43	−3.8	15	15.46	3.1
258								

273	Lactose	100	15	15.18	1.18	15	15.15	1
258								

273	Starch	100	15	14.9	−0.6	15	15.4	2.9
258								

273	NaHCO_3_	100	15	14.97	−0.2	15	15.36	2.8
258								

273	All of above	100	15	15.25	1.68	15	14.8	−1.3
258								

**Table 15:** Effect of interferences on the correction absorbance method.

*λ*	Type of interferences	Amount of interferences (*µ*g/mL)	HCT^*∗*^ (*µ*g/mL)			CAN (*µ*g/mL)		
			Add	Found	%*E*	Add	Found	%*E*
340	Polyethylene glycol	100	15	15.53	3.5	15	15.34	2.5
250								

340	Sucrose	100	15	15.24	1.6	15	15.2	1.3
250								

340	Lactose	100	15	14.4	−4	15	14.62	−2.5
250								

340	Starch	100	15	15.18	1.2	15	15.13	0.92
250								

340	NaHCO_3_	100	15	14.62	−2.5	15	15.34	2.3
250								

340	All of above	100	15	15.47	3.1	15	15.11	0.74
250								

^*∗*^^1^ calculated using the regression equation of *Y* = 0.0054*x* + 0.0067 and the HCT calibration curve at 340 nm. ^*∗*^^2^ calculated using the regression equation of *Y* = 0.0295*x* + 0.0648 and the HCT calibration curve at 250 nm.

**Table 16:** Statistical comparison between the HPSAM and HPLC.

No	Name	HCT milligram/tablet				CAN milligram/tablet				
		HPLC	HPSAM	%*E*	% recovery	HPLC	HPSAM	%*E*	% recovery	
1	Awacand	11.75	11.68	−0.6	100.6	14.98	15.05	0.48		99.52
2	Candex	12.07	12.08	0.083	99.917	15.89	15.79	−0.64		100.64
3	Atacand	11.92	11.99	0.59	99.41	15	14.83	−1.16		101.16

^*∗*^^1^ calculated using the regression equation of *Y* = 0.0247*x* + 0.0297 and the HCT calibration curve at 283 nm.

**Table 17:** Statistical comparison between the *Q*-absorption ratio method and HPLC.

NO	Name	HCT milligram/tablet				CAN milligram/tablet			
		HPLC	*Q*-Abs	%*E*	% recovery	HPLC	*Q*.Abs	%*E*	% recovery
1	Awacand	11.75	11.57	−1.45	98.47	14.98	15.14	1.1	101.0
2	Candex	12.07	12.1	0.22	100.2	15.89	15.9	0.17	100.06
3	Atacand	11.92	11.88	−0.33	99.66	15	15.02	0.13	100.13

**Table 18:** Statistical comparison between the correction absorbance method and HPLC.

NO	Name	HCT milligram/tablet				CAN milligram/tablet			
		HPLC	Correction method	%*E*	% recovery	HPLC	Correction method	%*E*	% recovery
1	Awacand	11.75	11.41	2.9	97.1	14.98	15.07	−0.6	100.6
2	Candex	12.07	12.56	−3.9	104.1	15.89	16.21	−2	102
3	Atacand	11.92	12.1	−1.5	101.5	15	14.4	4	96

^*∗*^^1^ calculated using the regression equation of *Y* = 0.0054*x* + 0.0067 and the HCT calibration curve at 340 nm. ^*∗*^^2^ calculated using the regression equation of *Y* = 0.0295*x* + 0.0648 and the HCT calibration curve at 250 nm.

**Table 19:** The review of the published work for simultaneous determination of drugs by different chemical methods.

No	Method	Drug	Linear range (*μ*g/ml)	RSD%	Recovery%	LOD (*μ*g/ml)	Reference
1	Spectrophotometric	CAN	2.5–50	1.19	99.0	0.55	[45]
		HCT	1–30	0.74	99.0	0.32	

2	UV-spectrophotometric	CAN	2–24	0.205	101.2	—	[46]
		HCT	2–24	0.154	99.2	—	

3	RP-HPLC	CAN	6.25–18.75	—	99.78	0.410	[47]
		HCT	8–24	—	100.64	0.699	

4	Proposed methods (HPSAM)	CAN	1–46	0.2061	98.84	0.48	—
		HCT	1–44	0.3084	98.9	0.46	

5	Proposed methods (*Q*-absorption ratio)	CAN	1–46	0.09338	99.93	0.88	—
		HCT	1–44	0.1813	99.2	0.76	

6	Proposed methods (correction absorbance)	CAN	1–46	0.2273	100.33		—
		HCT	1–44	0.2226	100.73		

## Data Availability

The data used to support the findings of this study are included within the article.
